# Bioactivity of *Ailanthus altissima* (Mill.) Swingle Extracts on Wheat Germination and Rice Weevil Survival

**DOI:** 10.3390/plants15081250

**Published:** 2026-04-18

**Authors:** Radenka Kolarov, Velemir Ninkovic, Sonja Gvozdenac, Dan Cristian Vodnar, Floricuta Ranga, Dejan Prvulović

**Affiliations:** 1 Department of Field and Vegetable Crops, Faculty of Agriculture, University of Novi Sad, 21000 Novi Sad, Serbia; radenka.kolarov@polj.uns.ac.rs (R.K.); dejan.prvulovic@polj.uns.ac.rs (D.P.); 2 Department of Ecology, Swedish University of Agricultural Sciences, 750 07 Uppsala, Sweden; 3 Institute of Field and Vegetable Crops, 21000 Novi Sad, Serbia; sonja.gvozdenac@ifvcns.ns.ac.rs; 4 Department of Food Science, University of Agricultural Sciences and Veterinary Medicine, 400372 Cluj-Napoca, Romania; dan.vodnar@usamvcluj.ro (D.C.V.); floricutza_ro@yahoo.com (F.R.)

**Keywords:** allelopathy, invasive species, secondary metabolites, seed priming, seed germination, *Sitophilus oryzae*, biopesticides

## Abstract

Invasive plant species are increasingly recognized not only as ecological threats but also as potential sources of bioactive compounds with agricultural applications. However, the combined allelopathic and insecticidal potential of *Ailanthus altissima’s* different plant parts remains insufficiently explored. This study evaluated the bioactivity of different plant part (leaf, bark, and branch) extracts of *A. altissima*. Secondary metabolites were characterized by HPLC–DAD–MS, while ethanol extracts (0.5–5%) were tested on wheat (*Triticum aestivum*) seed germination, seedling growth, oxidative status, and on the survival and repellency of the rice weevil (*Sitophilus oryzae*). Biological responses were strongly plant part and concentration-dependent. Leaf extracts contained the highest phenolic levels, dominated by caffeoylquinic acids and quercetin derivatives, whereas bark and branch extracts showed lower but compositionally distinct profiles. Despite this, bark and branch extracts produced the strongest biological effects, inhibiting germination energy and root growth at higher concentrations, while leaf extracts stimulated seedling performance, including increased vigor index, while in insect bioassays, bark and branch extracts caused higher mortality and stronger suppression of rice weevil populations. This study provides new evidence that biomass extracts of the invasive species *A. altissima* represent a promising source of biologically active compounds with both allelopathic and insecticidal properties, highlighting its potential valorization as a plant-based biopesticide for sustainable pest management.

## 1. Introduction

Invasive woody plants pose a significant ecological challenge globally, particularly in disturbed ecosystems that facilitate their rapid spread. These species are also being investigated as sources of biologically active compounds for sustainable agriculture [1,2]. Their success is due to ecological plasticity, efficient reproduction, and strong competitiveness, allowing them to thrive in fragmented and human-altered habitats [3,4,5]. Urban landscapes, characterized by frequent disturbance and reduced resilience, often promote the establishment and dominance of invasive woody species, leading to major changes in plant communities and ecosystem function [6,7].

Allelopathy is a key factor in the competitive success of invasive plants. It involves the production and release of secondary metabolites, or allelochemicals, which influence the germination, growth, and physiology of neighboring plants [8,9]. These compounds enter the environment through root exudation, volatilization, leaching, or decomposition of plant litter [10]. Allelochemicals can alter seed germination, root growth, nutrient uptake, and oxidative balance in nearby vegetation. These effects give invasive plants a competitive advantage and help them suppress other species, supporting their dominance in invaded ecosystems [11,12]. Secondary metabolites from invasive plants are increasingly studied as sources of biologically active compounds. Many invasive species contain phenolic acids, flavonoids, tannins, and other metabolites with phytotoxic, antimicrobial, and insecticidal properties [10,13]. This interest has led to the concept of invasive species biomass valorization, which explores using invasive plant material as a renewable resource for sustainable agricultural products. Valorizing invasive plant biomass addresses both invasive plant control and the development of alternatives to synthetic agrochemicals [14].

Using invasive plant biomass is especially relevant for stored wheat (*Triticum aestivum* L.), where both grain protection and seed viability are essential. Wheat seeds are highly susceptible to storage pests such as the rice weevil (*Sitophilus oryzae* L.), which causes significant losses during storage. Effective pest control must reduce insect populations while preserving seed germination and seedling development, emphasizing the value of plant-derived bioactive compounds that protect stored grain without harming seeds. Plant-derived bioactive compounds provide practical solutions for managing storage pests. Post-harvest insect infestations threaten global food security by causing substantial losses of stored grains [15,16]. The rice weevil is among the most destructive pests of stored cereals worldwide due to its rapid reproduction and ability to infest grains during storage [17]. Growing restrictions on synthetic pesticides, along with concerns about environmental contamination, human health, and insect resistance, have increased interest in natural pest management alternatives [18].

Plant-derived biopesticides are promising, especially when sourced from abundant and renewable plant resources [19]. Invasive woody species are particularly suitable due to their high biomass production and biochemical diversity [20]. Their secondary metabolites can affect plant growth and insect behavior depending on concentration: lower concentrations may stimulate growth or stress tolerance, while higher concentrations often have inhibitory or toxic effects. The distribution of these metabolites often varies among different plant parts, reflecting specific ecological functions and defense strategies [21,22,23,24]. Although interest in plant-derived allelochemicals and botanical insecticides is growing, few studies have evaluated both the phytotoxic and insecticidal effects of invasive woody species at the same time. The interactions between plant extracts used as seed treatments and their effects on storage pests are not well understood. Investigating these dual activities could reveal important ecological functions of invasive plants and identify new opportunities for sustainable crop protection.

*Ailanthus altissima* (Mill.) Swingle, or the tree of heaven, is among the most aggressive and widespread invasive woody species in Europe and other regions [23]. It is characterized by rapid growth, high reproductive capacity, and strong allelopathic effects on surrounding vegetation. *Ailanthus altissima* also produces a diverse range of secondary metabolites that may contribute to its ecological dominance.

This study aimed to identify the dominant secondary metabolites in ethanol extracts of different *A. altissima* plant parts, namely leaves, bark, and branches, and to evaluate their biological activities in relation to stored wheat protection. The allelopathic potential of the extracts, applied at different concentrations, will be assessed through wheat seed germination and early seedling growth in order to determine their effects on seed viability and initial plant development. In parallel, the insecticidal activities of the same extracts will be examined against the rice weevil, *S. oryzae*. By integrating metabolomic profiling with plant- and insect-based bioassays, the study sought to determine whether metabolites derived from this invasive species could simultaneously preserve desirable germination and seedling traits while suppressing insect populations, thereby supporting their potential use in sustainable grain protection. It was hypothesized that extracts obtained from leaves, bark, and branches would exhibit biologically relevant activity, with the magnitude and direction of the response depending on plant part and extract concentration. More specifically, the study was designed to test whether these extracts could reduce progeny production in *S. oryzae* while maintaining favorable wheat germination and early seedling performance, including growth, oxidative status, and phenolic-related traits.

## 2. Results

### 2.1. Phenolic Compounds of Ailanthus altissima Leaf, Bark and Branch Extracts

**Figure 1:**
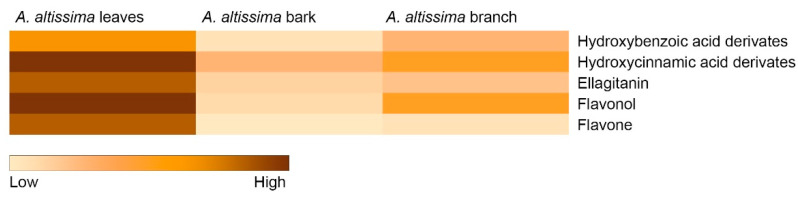
Heat map of phenolic compound classes in leaves, bark, and branches of *Ailanthus altissima*.

HPLC–DAD–MS analysis demonstrated a pronounced plant part used-specific distribution of phenolic compounds in *A. altissima*, with leaves representing the dominant reservoir of secondary metabolites (Table S1). In contrast, woody tissues exhibited markedly lower phenolic accumulation, indicating strong metabolic differentiation among plant parts. The phenolic composition of the leaves was characterized mainly by hydroxycinnamic acid derivatives and flavonol glycosides, which together constituted the dominant fraction of the detected metabolites. These compounds were present in significantly higher amounts in leaf extracts compared with bark and branch extracts (*p* < 0.05). Woody tissues contained only minor amounts of these metabolites and exhibited a simpler chemical profile. Certain phenolic acids, including ferulic and isoferulic acid derivatives, were detected primarily in woody tissue; however, their concentrations remained significantly lower than the dominant phenolic compounds detected in leaves. The plant part used-specific metabolic patterns were further supported by heat map visualization (Figure 1), which clearly illustrated the clustering of high phenolic abundance in leaf tissues across all detected phenolic classes, including hydroxybenzoic acids, hydroxycinnamic acids, ellagitannins, flavonols, and flavones. In contrast, bark extracts showed generally low signal intensities for most metabolite classes, whereas branch extracts displayed moderate levels of hydroxycinnamic derivatives but limited flavonoid representation. These results indicate that *A. altissima* preferentially allocates phenolic metabolites to leaves, which likely function as the primary biochemical interface with the environment and play a central role in mediating ecological interactions.

### 2.2. Germination and Early Growth Responses of Wheat to Ailanthus altissima Extracts

**Figure 2:**
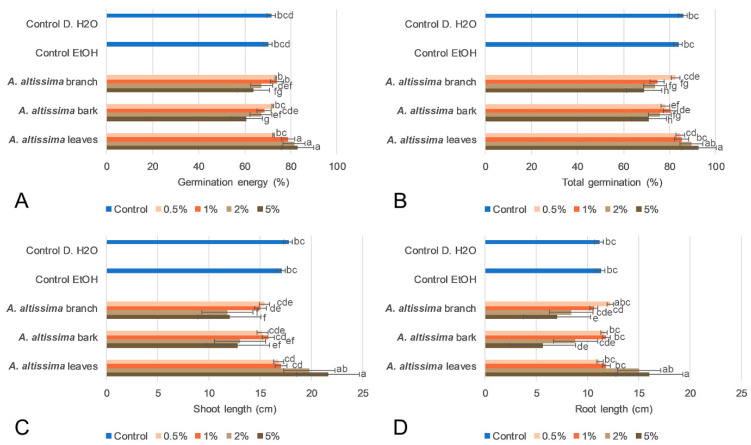
Effects of 70% ethanol extracts of *Ailanthus altissima* leaves, bark, and branches on seed germination and early seedling growth. (**A**) Germination energy, (**B**) germination percentage, (**C**) shoot length, and (**D**) root length of seedlings treated with *A. altissima* extracts at different concentrations (0.5%, 1%, 2%, and 5%), compared with the 70% ethanol and distilled water as a control. Values are presented as means ± SD. Different lowercase letters indicate statistically significant differences among treatments at *p* < 0.05.

Plant part and extract concentration significantly affected all measured parameters of wheat response, including germination energy, total germination, shoot length, and root length. Two-way ANOVA revealed significant effects of both factors, while a significant plant part × concentration interaction was also detected for several traits, indicating that the effect of extract concentration depended on the plant organ used for extraction (Table S2). Among the germination parameters, germination energy was one of the most sensitive indicators of extract activity (Figure 2). The strongest inhibitory effects occurred at the highest concentration (5%) of bark and branch extracts, which significantly reduced germination energy compared with the controls (*p* < 0.001, respectively) and most lower concentrations (Figure 2A). In contrast, leaf extracts remained largely comparable to the controls, indicating the absence of pronounced inhibitory effects. A similar trend was observed for total germination, although the differences were less pronounced than those recorded for germination energy (F_6,48_ = 28.1, *p* < 0.001) (Figure 2B). Higher concentrations of bark and branch extracts reduced total germination, whereas leaf extracts remained closer to the control values. This suggests that woody extracts mainly affected the speed and uniformity of germination, while the final germination percentage was less strongly influenced. Differences among plant parts became even more evident during early seedling development. Shoot length was significantly reduced by higher concentrations of bark and branch extracts, while leaf extracts showed weaker effects and, in several cases, remained statistically similar to the controls (F_6,48_ = 15.7, *p* < 0.001) (Table S2) (Figure 2C). Root length was the most sensitive morphological parameter, showing the strongest inhibition in response to the 5% bark and branch extracts (F_6,48_ = 14.1, *p* < 0.001) (Figure 2D). In contrast, leaf extracts produced neutral or slightly stimulatory effects at lower concentrations. Overall, the results indicate that wheat’s phytotoxic response depended on both the origin of the extract and its concentration. The strongest inhibitory effects were associated mainly with extracts obtained from woody tissues, especially at higher concentrations. Although leaf tissues contained higher total phenolic content, their extracts showed weaker phytotoxicity, suggesting that biological effects were more closely related to qualitative differences in metabolite composition than to total phenolic content alone.

**Figure 3:**
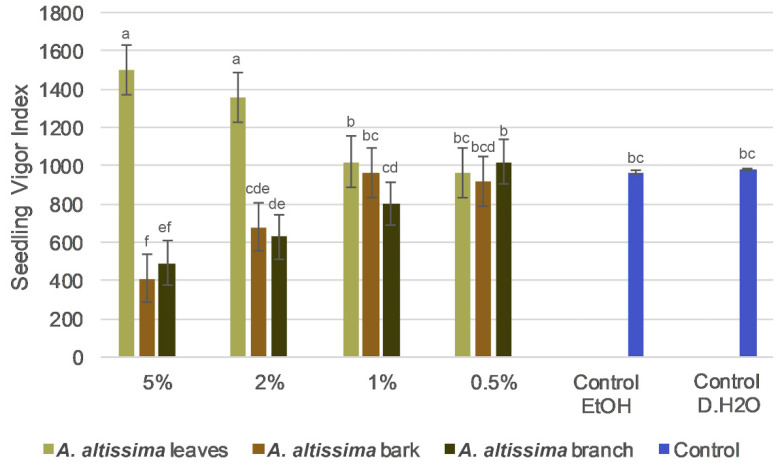
Concentration-dependent effects of 70% ethanol extracts of *Ailanthus altissima* leaves, bark, and branches on wheat seed Vigor index compared with the 70% ethanol and distilled water as a control. Values are presented as means ± SD. Different lowercase letters indicate statistically significant differences among treatments at *p* < 0.05.

The seedling vigor index showed plant part used- and concentration-dependent preference to *A. altissima* extracts (Figure 3). Significant differences were observed among plant parts and concentration (F_6,48_ = 22.5, *p* < 0.001) (Table S3). At the highest concentration (5%), leaf extracts had the highest vigor index values, significantly exceeding the control and the bark and branch treatments. In contrast, bark and branch extracts resulted in markedly lower vigor indices at this concentration, indicating strong inhibitory effects on early seedling development. A similar, but less pronounced, pattern was observed at 2%, with leaf extracts showing higher vigor values than woody tissues. At lower concentrations (1% and 0.5%), differences among treatments were reduced. Overall, leaf extracts showed a concentration-dependent increase in vigor, suggesting a mild stimulatory effect, whereas bark and branch extracts caused a progressive decline in seedling vigor with increasing concentration.

The concentrations of total phenolics, tannins, and flavonoids in wheat seedlings were significantly influenced by both the extract concentration and the plant part used for seed treatment. Two-way ANOVA revealed a significant plant part and concentration interaction for all three polyphenolic contents (Table S4). The contents of total phenolics, tannins, and flavonoids in wheat seedlings were strongly affected by both extract concentration and the plant part used for seed treatment. Regarding total phenolic content, the significance of plant part (F_2,60_ = 323.2, *p* < 0.001) was less pronounced than the concentration effect (F_2,60_ = 717.3, *p* < 0.001), which was more statistically pronounced. The same trend was observed in both total tannins (plant part F_2,60_ = 717.3, *p* < 0.001; concentration F_2,60_ = 1145.2, *p* < 0.001) and total flavonoid content (plant part F_2,60_ = 121.4, *p* < 0.001; concentration F_2,60_ = 1151.2, *p* < 0.001).

At 5%, leaf treatments significantly increased the levels of total phenolics, tannins, and flavonoids compared with bark and branch treatments, as well as both controls, and a similar trend was observed at 2%, where leaf extracts still produced significantly higher metabolite levels than woody tissues (Table 1). At lower extract concentrations (1% and 0.5%), differences among plant parts used as biopreparates were lower, indicating a weaker biochemical response. In conclusion, increasing extract concentration resulted in a progressive increase in phenolic, tannin, and flavonoid accumulation in wheat seedlings. The strongest induction was consistently marked with leaf extracts, suggesting that these extracts elicited a more pronounced metabolic response in seedlings than bark or branch extracts.

Exposure of plants to bioactive secondary metabolites can induce oxidative stress by generating reactive oxygen species, underscoring the importance of evaluating antioxidant responses for understanding plant defense mechanisms. Therefore, the antioxidant capacity of wheat seedlings derived from seeds treated with *A. altissima* extracts was evaluated using three complementary assays, DPPH, ABTS, and FRAP. Antioxidant assays showed that wheat seedlings derived from seeds treated with *A. altissima* extracts exhibited significantly enhanced antioxidant capacity, with responses strongly dependent on both extract concentration and the plant part used (Table S5). DPPH assay showed that the plant part used was more significant than the concentration (plant part F_2,60_ = 1887.01, *p* < 0.001; concentration F_2,60_ = 1150.1, *p* < 0.001), while ABTS (plant part F_2,60_ = 1354.8, *p* < 0.001; concentration F_2,60_ = 1619.5, *p* < 0.001) and FRAP (plant part F_2,60_ = 1217.9, *p* < 0.001; concentration F_2,60_ = 1576.5, *p* < 0.001) assays showed the same trend, indicating that extract concentration was more significant.

Comparisons were performed among extracts at the same concentration (e.g., leaf vs. bark vs. branch vs. controls at 5%, 2%, 1%, and 0.5%; Table 2). At 5%, seedlings derived from seeds treated with leaf extracts displayed the highest antioxidant responses across all assays, with significantly higher DPPH, ABTS, and FRAP values compared with bark, branch, and control treatments. Bark and branch extracts also increased antioxidant activity relative to controls, but to a significantly lower extent. A similar trend was observed at 2%, whereas differences among treatments at 1% and 0.5% were smaller and not always statistically significant compared with the controls used. The elevated DPPH, ABTS, and FRAP values indicate enhanced radical-scavenging capacity and reducing power in seedlings from treated seeds. Notably, the strongest antioxidant responses occurred with treatments using leaf extracts, which also had the highest phenolic content, suggesting that phenolic secondary metabolites contribute to enhanced antioxidant capacity.

Seed treatment with different *A. altissima* extracts significantly affected the antioxidant enzyme system of wheat seedlings (Table 3). The activities of SOD, CAT, GPX, POD, and APX were significantly influenced by both plant part and extract concentration for all parameters. Even though plant parts and concentration themselves were significant factors, significance was not observed when both were included, except for SOD (F_2,60_ = 199.2, *p* < 0.001). Overall, enzyme activities showed a clear concentration-dependent pattern (Table S6); thus, the results confirm a pronounced concentration-dependent modulation of antioxidant enzyme activity in wheat seedlings.

Lipid peroxidation analysis indicated only moderate membrane oxidation in wheat seedlings after seed treatment with *A. altissima* extracts (Table 4). LP values increased slightly at the highest concentration (F_2,60_ = 488.1, *p* < 0.001) (Table S7) compared to the controls used, but remained significantly lower than levels associated with severe oxidative stress (Table 4). At 2%, lipid peroxidation decreased, while at 1% and 0.5%, values were close to the controls, and differences were mostly not significant. The relatively low LP levels suggest that the extracts did not induce severe membrane damage but rather triggered a controlled oxidative signal. This response was associated with increased antioxidant capacity and enhanced activity of antioxidant enzymes, signaling efficient redox regulation and maintenance of cellular stability in wheat seedlings.

### 2.3. Insecticidal Effect on Storage Pest Rice Weevil

**Figure 4:**
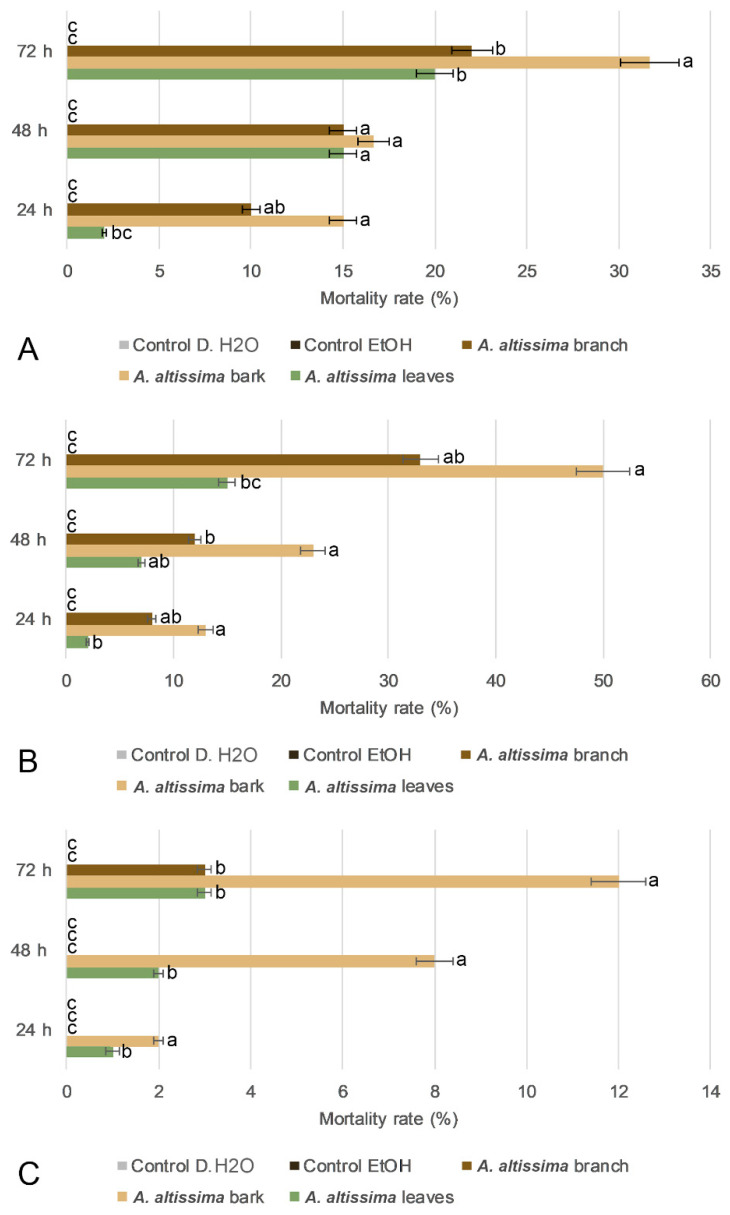
Effects of 70% ethanol extracts of *Ailanthus altissima* leaves, bark, and branches on mortality of the rice weevil adults after 24, 48, and 72 h of exposure. Panels (**A**–**C**) correspond to extract concentrations of 5% (**A**), 2% (**B**), and 1% (**C**), while 0.5% showed no effect, respectively. Panels show mortality rates (%) recorded at 24, 48, and 72 h after exposure. Treatments were compared with 70% ethanol and distilled water controls. Values are presented as means ± SD. Different lowercase letters indicate statistically significant differences among treatments at *p* < 0.05.

Plant-derived extracts, an important source of bioactive compounds, were evaluated against the major stored-grain pest, *S. oryzae*, to assess their potential for sustainable pest management. Insect bioassays showed that adult mortality was significantly affected by plant part, extract concentration, exposure time, and all corresponding interactions (repeated-measurements) (Table S8). Panels A–C represent extract concentrations of 5%, 2%, and 1%, respectively, whereas both control treatments (distilled water and ethanol) consistently exhibited the lowest mortality and were generally assigned to the lowest significance group (“c”) across exposure periods. At 5% (Figure 4A), mortality increased significantly with exposure time, reaching a maximum at 72 h. Bark extract produced the strongest insecticidal effect, showing significantly higher mortality than the other treatments, whereas branch and leaf extracts displayed lower but still detectable activity. A similar pattern was observed at 2% (Figure 4B), where bark extract remained the most effective treatment, while branch extract showed intermediate activity and leaf extract produced the weakest response among the plant-derived treatments. At 1% (Figure 4C), overall mortality was substantially reduced, although bark extract still maintained the highest activity compared with the other plant parts. Collectively, these findings demonstrate a clear concentration- and time-dependent insecticidal effect of *A. altissima* extracts against *S. oryzae*, with bark extract showing the greatest efficacy.

**Figure 5:**
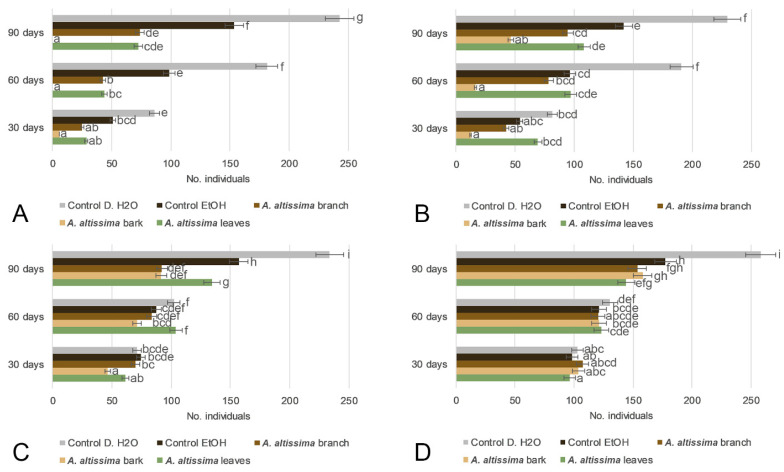
Effects of 70% ethanol extracts of *Ailanthus altissima* leaves, bark, and branches on the rice weevil progeny production after 90 days. Panels (**A**–**D**) correspond to extract concentrations of 5% (**A**), 2% (**B**), 1% (**C**), and 0.5% (**D**). Present changes in the number of individuals were monitored at 7, 30, 60, and 90 days. Treatments were compared with ethanol and distilled water controls. Values are presented as means ± SD. Different lowercase letters indicate statistically significant differences among treatments at *p* < 0.05.

Following the acute insecticidal effects observed in the short-term bioassay (Figure 4), the longer-term impact of *A. altissima* extracts on *S. oryzae* population development was evaluated by monitoring progeny emergence at 30, 60, and 90 days (Figure 5). Repeated-measurements ANOVA revealed that progeny production was significantly affected by plant part, extract concentration, time, and all corresponding interactions (F_24,80_ = 5.1, *p* < 0.001), whereas the DAYS × Concentration interaction was also significant, although less pronounced (F_6,80_ = 3.7, *p* = 0.002678) (Table S9). At 5% (Figure 5A), bark and branch extracts exerted the strongest suppressive effects on progeny production, with bark treatment resulting in complete inhibition of adult emergence after 90 days. Leaf extract also reduced progeny emergence relative to both control treatments, although to a lesser extent. In contrast, distilled water and ethanol controls consistently showed the highest numbers of emerged adults throughout the experimental period, indicating unrestricted population development. At 2% (Figure 4B), progeny production remained clearly lower than in the controls, but the inhibitory effect was weaker than that observed at 5%, confirming a concentration-dependent reduction in reproductive suppression. At 1% and 0.5% (Figure 5C,D), the number of emerged adults increased further and approached control levels, indicating a substantial decline in treatment efficacy at lower concentrations. Overall, these findings demonstrate that *A. altissima* extracts, particularly those derived from woody tissues, significantly suppressed the long-term population development of *S. oryzae* in a concentration- and time-dependent manner, with bark extract showing the greatest efficacy.

## 3. Discussion

*Ailanthus altissima* extracts used as biopreparates exhibit plant part specific, concentration-dependent biological activities in both wheat and pest storage insect bioassays. Our study shows that leaf extracts at 5% and 2% have the greatest stimulatory effect in the wheat germination assay, while bark and branch extracts inhibit germination energy, total germination, and seedling development at the same concentrations. This is the first report of the effect of *A. altissima* extracts, and this is of key importance because the impact on wheat seeds must remain undisturbed if biopreparations of *A. altissima* extracts are to be used with the aim of progeny production of *S. oryzae* as a pest storage insect of great importance.

The present study was conceived to assess the biological activity of whole *A. altissima* extracts as chemically complex mixtures of secondary metabolites rather than to identify a single dominant active principle. This distinction is particularly relevant because the activity of plant extracts frequently arises from the combined effects of multiple constituents, including additive and synergistic interactions, whereas isolated compounds do not necessarily reproduce the biological properties of the crude extract. In this context, the metabolomic analysis enabled the identification of several compounds potentially associated with the observed biological responses, which should therefore be interpreted as candidate contributors to the overall allelopathic and insecticidal activity rather than as conclusively confirmed active agents. Specifically, quercetin-glucoside, cryptochlorogenic acid, neochlorogenic acid, chlorogenic acid, and quercetin-malonyl-glucoside were associated with the distinct bioactivities observed among plant parts, whereas ferulic acid and isoferulic acid, particularly abundant in bark and branch extracts, were linked to the more pronounced phytotoxic and insecticidal effects. Results indicate that the bioactivity of *A. altissima* extracts is governed primarily by tissue-specific metabolite composition, while the observed effects most likely depend on the integrated action of multiple constituents. Other secondary metabolites likely also contribute to the observed effects of the extracts, while the absence of a clear dose–response relationship suggests that these outcomes arise from complex interactions among multiple bioactive compounds [10]. Hydroxycinnamic acid derivatives and flavonol glycosides in leaves are associated with redox modulation [24,25,26]. A study on *Eupatorium adenophorum* allelopathy in *Arabidopsis thaliana* demonstrated significant shifts in primary and secondary metabolites, including those involved in energy metabolism and amino acid biosynthesis, indicating that phytotoxicity is caused by specific metabolites rather than general phenolic content [27].

As demonstrated in the present study, the application of pre-sowing priming seed treatment can enhance germination, seedling vitality, and stress tolerance. However, the effects of these exudates may vary depending on their plant part of origin and concentration, and in some cases they may exert inhibitory or phytotoxic effects on both the plant and seed consumers. Although the leaves of *A. altissima* contained higher overall phenolic levels than bark and branch extracts, this did not correspond to stronger effects on wheat germination or in the storage pest bioassay. Instead, extracts derived from bark and branches had a more pronounced effect on germination energy and root growth, especially at higher concentrations. Clear plant part-specific differences in phytotoxic effects were observed in wheat, with roots showing the greatest susceptibility. This pattern suggests interference with early cell division and key biochemical processes during seedling establishment. Similar plant part used and composition-dependent effects have also been reported in *Ficus carica* across different cultivars [28].

Root growth proved to be the most sensitive morphological parameter, showing significant decreases in root elongation and overall length in wheat seedlings exposed to bark and branch extracts of *A. altissima*, especially at higher concentrations when it comes to bark and branch extracts. This aligns with existing knowledge that roots are the primary exposure site for allelochemicals and react quickly to changes in membrane integrity, oxidative state, and hormone regulation. According to Cheng and Cheng [29], allelochemicals often disrupt root meristem activity by inhibiting cell division and elongation, causing chromosomal abnormalities, and altering microtubule organization. Compounds such as benzoxazinoids and phenolic acids can lower the mitotic index and impair tubulin dynamics, thus suppressing radicle growth.

In our experiment, wheat seedlings treated with the *A. altissima* extracts exhibited a moderate but significant increase in lipid peroxidation compared to the control group. This increase, accompanied by activation of antioxidant enzymes such as SOD, CAT, GPX, and APX, indicates that the treatment induced oxidative stress, which was counteracted by the activation of the plant’s antioxidant defense system. Strong activation of key antioxidant enzymes and elevated non-enzymatic antioxidant capacity were also observed in treated seedlings. The enhanced activity of antioxidant enzymes, along with the significant increase in non-enzymatic antioxidant capacity tests, explains the higher radical-scavenging activity and improved oxidative stress-reducing power. This phenomenon is indicative of enhanced regulation of reactive oxygen species and maintenance of redox homeostasis. The increased total phenolic content in *A. altissima* extracts indicates that the extracts positively stimulate the accumulation of these molecules in wheat seedlings.

Extracts from bark and branch exhibited a more pronounced insecticidal effect on the survival of rice weevils after 24, 48 and 72 h, as well as a more evident long-term suppression of population development, as evidenced by reduced progeny production. In contrast, leaf extracts had less significant results on *S. oryzae* progeny production. These results suggest that woody tissues may accumulate defense-related metabolites with inhibitory properties. These results support the notion that plant-derived bioactive compounds can cause both acute mortality and long-term physiological impairment in insects, effects that have not previously been reported for *A. altissima* extracts. The effects on population decline (reduction in progeny production) after 90 days indicate potential sublethal effects. Our previous study demonstrated that prolonged exposure to sublethal concentrations of leaf extracts was associated with reduced reproductive capacity, delayed development, altered feeding behavior, and disruption of detoxification and oxidative balance mechanisms in storage insects [30]. Repeated applications of such extracts could suppress pest populations across multiple generations by reducing reproductive output, thereby decreasing population pressure and crop losses over time. This approach aligns with an integrated pest management strategy, where reductions in pest population growth and survivorship are essential for sustained control [31]. Utilizing *A. altissima* extracts to induce population-level suppression could therefore offer tangible benefits for farmers seeking alternatives to synthetic insecticides.

The present findings may also be interpreted within a broader ecological context, as *Ailanthus altissima* appears to share important chemical defense traits with other invasive plant species known for both allelopathic and insecticidal activity. Similar dual bioactivity has been reported for *Parthenium hysterophorus*, whose extracts and secondary metabolites have been associated with strong phytotoxic effects on seed germination and early seedling growth, as well as insecticidal and larvicidal activity attributed largely to sesquiterpene lactones such as parthenin [32]. Comparable patterns have also been described for *Lantana camara*, an invasive species rich in phenolics, flavonoids, terpenoids, and related bioactive constituents, with documented allelopathic effects on neighboring plants and insecticidal potential against several pest species [33,34,35]. Likewise, *Chromolaena odorata* has been recognized as an invasive species in which secondary metabolites contribute both to allelopathic interference and to defensive or biocidal functions, reinforcing the view that invasion success is often supported by multifunctional chemical traits [34,36]. In the case of *Ambrosia artemisiifolia*, sesquiterpenes and other metabolites have also been linked to allelopathic activity, further supporting the idea that invasive plants frequently rely on chemically mediated interference with surrounding organisms [37]. Within this comparative framework, the results for *A. altissima* support the interpretation that its allelopathic and insecticidal effects are part of a broader chemically mediated defense syndrome commonly observed in successful invaders. At the same time, the tissue-specific metabolite patterns identified here highlight the originality of *A. altissima*, suggesting that although it shares this general ecological strategy with other invasive species, the specific composition of active metabolite mixtures differs among taxa and plant parts. The results of this study strongly support our hypothesis that ethanol extracts obtained from different *A. altissima* plant parts exhibit biologically relevant effects that vary according to both tissue origin and concentration. Distinct responses observed in wheat and *S. oryzae* bioassays indicate that leaves, bark, and branches differ not only in phytochemical composition but also in their functional biological roles. Leaf extracts, particularly at 5% and 2%, promoted wheat germination and improved early seedling performance, whereas bark and branch extracts showed comparatively stronger inhibitory effects on germination and growth, together with pronounced insecticidal activity, especially under prolonged exposure. Importantly, although woody extracts caused moderate reductions in germination and early growth at higher concentrations, these effects were not severe enough to be considered agronomically restrictive under the tested conditions. Taken together, these findings demonstrate a clear functional specialization among *A. altissima* plant parts and show that this invasive species represents a promising source of bioactive metabolites with dual relevance for stored grain systems. Specifically, the results suggest that selected extracts can suppress *S. oryzae* populations while preserving acceptable wheat germination and seedling performance, highlighting their potential for application in sustainable grain protection strategies.

## 4. Materials and Methods

### 4.1. Plant Material and Preparation of Extracts

**Figure 6:**
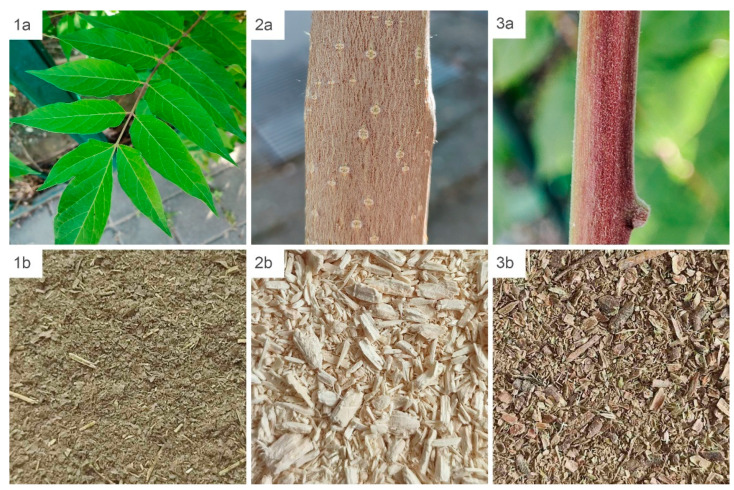
Plant material of *Ailanthus altissima* used for extract preparation: fresh leaves (**1a**), bark (**2a**), and branches (**3a**), and the corresponding dried and ground leaf (**1b**), bark (**2b**), and branch (**3b**) samples.

Plant material of *A. altissima* was collected from the quay area in Novi Sad, Serbia (45°14′51.9″ N, 19°51′22.1″ E). The collected material was separated according to plant part and processed for further extraction and analysis. For documentation purposes, a voucher copy of the material used in this study was deposited in the botanical collection of the Provincial Institute for Nature Protection. Leaves, bark, and branches were sampled from healthy, mature trees during the growing season. Bark and branch samples were obtained from two-year-old woody tissues, while fully developed leaves of intermediate physiological age were collected from the middle canopy zone (Figure 6). To minimize variability associated with microenvironmental conditions and tissue developmental stage, sampling was performed at a medium crown height of the species, and plant material was collected from the lateral sides of the trees where growth conditions were relatively uniform.

Prior to extraction, the collected plant material was dried in a laboratory oven (Memmert, Schwabach, Germany) at 40 °C under controlled conditions until a constant mass was achieved. Although the initial moisture content was not determined analytically, the same drying procedure was applied to all samples in order to ensure consistency and comparability among plant parts. After drying, the plant material was ground to a fine powder in a laboratory mill (Retsch GmbH, Haan, Germany). Ethanol extracts were prepared by macerating the powdered plant material with 70% ethanol at a 1:10 (*w*/*v*) ratio for 24 h at room temperature [38]. After extraction, the solutions were filtered and diluted with 70% ethanol to obtain final concentrations of 0.5%, 1%, 2%, and 5%. Distilled water and 70% ethanol were used as control treatments.

### 4.2. Secondary Metabolite Profiling

Phenolic compounds were extracted from 1 g of dried plant material using 10 mL of 70% ethanol. Samples were vortexed for 1 min, sonicated for 60 min at room temperature, and centrifuged at 10,000 rpm for 10 min. Supernatants were filtered through 0.45 μm nylon membrane filters, and 20 μL of each extract was injected into the HPLC system. Chromatographic analysis was performed using an HP 1200 liquid chromatograph equipped with a quaternary pump, autosampler, diode-array detector (DAD), and single-quadrupole mass spectrometer (Agilent Technologies, Santa Clara, CA, USA). Separation was achieved on a Kinetex XB-C18 column (5 μm, 4.6 × 150 mm; Phenomenex, Torrance, CA, USA) at 25 °C. The mobile phase consisted of water (A) and acetonitrile (B), both acidified with 0.1% acetic acid, and a linear gradient from 5% to 90% B was applied. The flow rate was 0.5 mL/min, and the total runtime was 30 min. Detection was performed by DAD at 280 and 340 nm and by mass spectrometry in positive-ion mode (*m*/*z* 120–1500). Phenolic compounds were identified by comparing retention times, UV spectra, and mass spectral data with authentic standards [39]. Data acquisition and processing were performed using Agilent ChemStation software. HPLC-grade acetonitrile and analytical reagents were obtained from Merck, Darmstadt, (Germany). Ultrapure water was produced using a Direct-Q UV purification system (Millipore, Burlington, MA, USA). Reference standards (gallic acid, chlorogenic acid, catechin, luteolin, and rutin; purity ≥ 99%) were obtained from Sigma-Aldrich, St. Louis, MO, USA.

### 4.3. Allelopathic Bioassay on Wheat Seed Germination

Allelopathic activity of *A. altissima* extracts was evaluated using wheat seeds. Wheat seeds of the cultivar “Zvezdana” were obtained from the Institute of Field and Vegetable Crops, Novi Sad, Serbia. Seeds were surface-sterilized and treated with extract solutions (0.5%, 1%, 2%, and 5%) for 10 min, then air-dried. Surface-sterilized seeds were treated for 10 min with *A. altissima* extract solutions at concentrations of 0.5%, 1%, 2%, and 5%, as well as the corresponding controls with distilled water and 70% ethanol. Treated seeds were then placed in sterile glass Petri dishes (150 mm in diameter) lined with two layers of filter paper and incubated in a growth chamber under controlled environmental conditions at 25 ± 1 °C, with a 12 h light/12 h dark photoperiod, light intensity of approximately 120–150 μmol m^−2^ s^−1^, and relative humidity of 65–70%. Germination energy was assessed on day 4 as the percentage of seeds that had produced a visibly emerged radicle of at least 2 mm, following the radicle-emergence criterion commonly used in seed testing [40]. Final germination percentage, shoot length, root length, and seedling vigor index were determined after 7 days according to standard seed testing procedures. Each treatment consisted of five replicates of 100 seeds, and the experiment was conducted in a completely randomized design.

### 4.4. Determination of Phenolic Compounds

Total phenolic and tannin contents in wheat seedling extracts were determined spectrophotometrically using the Folin–Ciocalteu method according to established protocols [41]. For total phenolic content, 3.4 mL of distilled water and 200 μL of Folin–Ciocalteu reagent were added to 20 μL of sample. After 5 min, 400 μL of sodium carbonate (20% *w*/*v* Na_2_CO_3_) solution was added, and the mixture was incubated for 60 min at room temperature. Total tannin content was determined indirectly after precipitation with polyvinylpolypyrrolidone (PVPP). Briefly, 0.1 g PVPP was mixed with 1 mL of distilled water and 1 mL of extract. The supernatant, containing non-tannin phenolics, was then analyzed using the same Folin–Ciocalteu procedure. Total tannin content was calculated as the difference between total phenolics and non-tannin phenolics and expressed as mg gallic acid equivalents per g dry weight (mg GAE/g DW) [42]. Absorbance was measured at 730 nm using a spectrophotometer (Thermo Scientific Evolution 220, Madison, WI, USA). A calibration curve was constructed using a series of gallic acid standard solutions. Total flavonoid content was determined using the aluminum chloride colorimetric method [43] based on the ability of flavonoids to form stable complexes with Al^3+^ ions. Briefly, the reaction mixture consisted of 0.5 mL of distilled water, 1.5 mL of AlCl_3_ solution (5% *w*/*v* in 70% ethanol), and 200 μL of the extract. After incubation for 15 min at room temperature, absorbance was measured at 430 nm using a spectrophotometer. A calibration curve was constructed using quercetin standard solutions. Results for phenolic compounds are expressed as mg quercetin equivalents per g dry weight (mg QE/g DW).

### 4.5. Determination of Antioxidant Activity

Antioxidant activity of wheat seedling extracts was evaluated using DPPH radical scavenging, ABTS radical cation decolorization, and ferric reducing antioxidant power (FRAP) assays. The FRAP assay was performed by mixing 10 μL of sample with 2 mL of FRAP reagent and measuring absorbance at 593 nm after 30 min. The FRAP reagent was prepared by mixing 300 mmol/L acetate buffer pH 3.6, 10 mmol/L TPTZ solution in 40 mmol/L HCl, and 20 mmol/L FeCl_3_·6H_2_O solution in a 10:1:1 ratio [44]. DPPH radical scavenging activity was determined by adding 3 mL of DPPH solution and 10 μL of sample, with absorbance measured at 517 nm after 30 min [45]. ABTS radical scavenging activity was assessed by mixing 3 mL of ABTS solution with 20 μL of sample and measuring absorbance at 734 nm [45,46]. Calibration curves for all assays were prepared using Trolox standard solutions, and antioxidant capacity was expressed as mg Trolox equivalents per g dry weight (mg TE/g DW). Antioxidant capacity was expressed as mg Trolox equivalents per g dry weight (mg TE/g DW).

### 4.6. Determination of Enzyme Activity

Activities of antioxidant enzymes, including superoxide dismutase, (23 °C, buffer pH 7.8) (SOD) [47], catalase (23 °C, buffer pH 7.0) (CAT) [48], guaiacol peroxidase (GPX) [49], pyrogallol peroxidase (POD) [50], and ascorbate peroxidase (APX) [51], were determined spectrophotometrically according to established protocols. For each assay, 50–100 μL of enzyme extract was added to the corresponding reaction mixtures containing the appropriate buffers and substrates, and the total reaction volume was adjusted to 3 mL. SOD activity was determined by monitoring the inhibition of nitroblue tetrazolium (NBT) reduction at 560 nm. CAT activity was measured based on the decomposition of hydrogen peroxide (H_2_O_2_) at 240 nm. GPX activity was determined using guaiacol as a substrate by monitoring the increase in absorbance of tetraguaiacol at 470 nm, while POD activity was measured using pyrogallol as a substrate at 430 nm. APX activity was determined by following the oxidation of ascorbate in the presence of hydrogen peroxide at 290 nm. Results are expressed as U per mg of protein (Umg/protein).

### 4.7. Insecticidal Activity Assay

The insecticidal activity of *A. altissima* extracts was evaluated against adult *S. oryzae* using a contact–digestive bioassay on treated wheat seeds following established protocols. Wheat seeds were treated with extract solutions at 0.5%, 1%, 2%, and 5% concentrations, air-dried, and subsequently placed in Petri dishes containing 20 g of wheat grain. Each treatment was conducted in three replicates, with 20 adult *S. oryzae* individuals (mixed sex) per experimental unit, and results were compared with distilled water and 70% ethanol controls [52]. The insects used in the study were obtained from a previously established colony. Adult insects were introduced to the treated seeds and maintained under controlled laboratory conditions (25 ± 1 °C, 65–70% relative humidity, and a 12 h light/12 h dark photoperiod). Insect mortality was recorded at 24, 48, and 72 h, and insects were considered dead if they showed no movement when gently prodded. Natural mortality was corrected using Abbott’s formula:

E=(C−T)/C×100

E—corrected mortality (%);

C—mortality in the control group (%);

T—mortality in the treated group (%).

To assess long-term population development, the parental adults were not removed from the experimental units, and newly emerged adults (progeny production) were counted after 30, 60, and 90 days.

### 4.8. Statistical Analysis

Statistical analyses were carried out using STATISTICA software v14.1.8 (TIBCO Software Inc., Palo Alto, CA, USA). The normality of data distribution was tested using the Shapiro–Wilk test, and homogeneity of variances was assessed by Levene’s test. All parameters related to the effects of plant part and extract concentration were analyzed by two-way analysis of variance (ANOVA), with plant part and extract concentration included as fixed factors, in order to determine their main effects and interaction. In the case of insect bioassay data, repeated-measures ANOVA was used, since the same experimental units were evaluated over multiple time points. When the ANOVA revealed significant effects, differences among means were determined using Tukey’s honestly significant difference (HSD) test. Statistical significance was accepted at *p* < 0.05. The heat map was prepared in Microsoft Excel from the relative abundance data for identified secondary metabolites presented in Supplementary Table S1.

## Figures and Tables

**Table 1:** Total phenolic, tannin, and flavonoid content in wheat seedlings following treatment with *Ailanthus altissima* extracts.

	Total Phenolics	Total Tannins	Total Flavonoids
*A. altissima* leaves 5%	18.32 ± 0.73 ^a^	13.40 ± 0.55 ^a^	15.17 ± 0.76 ^a^
*A. altissima* bark 5%	12.62 ± 0.59 ^b^	8.05 ± 0.33 ^b^	8.49 ± 0.47 ^b^
*A. altissima* branch 5%	8.23 ± 0.18 ^c^	7.84 ± 0.38 ^b^	9.17 ± 0.36 ^b^
Control EtOH	4.61 ± 0.22 ^d^	4.64 ± 0.44 ^c^	2.96 ± 0.15 ^c^
Control D. H20	3.20 ± 0.19 ^e^	3.02 ± 0.14 ^d^	2.12 ± 0.15 ^d^
*A. altissima* leaves 2%	16.13 ± 0.64 ^a^	7.26 ± 0.30 ^a^	7.86 ± 0.52 ^ab^
*A. altissima* bark 2%	11.21 ± 0.49 ^c^	6.68 ± 0.36 ^a^	7.52 ± 0.19 ^b^
*A. altissima* branch 2%	12.24 ± 0.43 ^b^	7.51 ± 0.16 ^b^	8.17 ± 0.19 ^a^
Control EtOH	4.49 ± 0.38 ^d^	2.69 ± 0.10 ^c^	3.02 ± 0.22 ^c^
Control D. H20	3.08 ± 0.24 ^e^	1.93 ± 0.16 ^d^	2.10 ± 0.06 ^d^
*A. altissima* leaves 1%	10.02 ± 1.09 ^a^	4.26 ± 0.22 ^a^	5.00 ± 0.20 ^a^
*A. altissima* bark 1%	6.91 ± 0.62 ^b^	4.29 ± 0.27 ^a^	4.79 ± 0.32 ^a^
*A. altissima* branch 1%	7.41 ± 0.38 ^b^	4.62 ± 0.60 ^a^	5.00 ± 0.33 ^a^
Control EtOH	4.46 ± 0.33 ^c^	2.69 ± 0.15 ^b^	3.00 ± 0.12 ^b^
Control D. H20	3.24 ± 0.17 ^d^	1.85 ± 0.12 ^c^	2.15 ± 0.10 ^c^
*A. altissima* leaves 0.5%	5.50 ± 0.51 ^a^	3.17 ± 0.34 ^b^	3.40 ± 0.25 ^b^
*A. altissima* bark 0.5%	5.31 ± 0.76 ^a^	3.08 ± 0.20 ^b^	3.59 ± 0.26 ^b^
*A. altissima* branch 0.5%	5.63 ± 0.44 ^a^	3.40 ± 0.47 ^b^	3.60 ± 0.17 ^b^
Control EtOH	4.40 ± 0.26 ^b^	4.70 ± 0.16 ^a^	2.98 ± 0.13 ^a^
Control D. H20	4.42 ± 0.20 ^b^	3.11 ± 0.31 ^b^	2.05 ± 0.07 ^b^

Values are presented as means ± SD. Different lowercase letters indicate statistically significant differences among treatments at *p* < 0.05. Results are expressed as mg gallic acid equivalents/g of dry weight (mg GAE/g DW) for total phenolics and total tannins, while for total flavonoids, mg quercetin equivalents/g of dry weight (mg QE/g DW).

**Table 2:** Non-enzymatic antioxidant capacity of wheat seedlings treated with *Ailanthus altissima* extracts.

	DPPH	ABTS	FRAP
*A. altissima* leaves 5%	199.90 ± 10.67 ^a^	138.91 ± 5.96 ^a^	3.60 ± 0.19 ^a^
*A. altissima* bark 5%	33.16 ± 1.25 ^b^	47.13 ± 1.57 ^b^	1.25 ± 0.02 ^b^
*A. altissima* branch 5%	35.65 ± 1.34 ^b^	50.54 ± 1.06 ^b^	1.31 ± 0.05 ^b^
Control EtOH	9.86 ± 0.61 ^c^	13.68 ± 1.16 ^c^	0.35 ± 0.03 ^c^
Control D. H20	4.37 ± 0.23 ^c^	5.85 ± 0.39 ^d^	0.15 ± 0.01 ^d^
*A. altissima* leaves 2%	167.02 ± 10.34 ^a^	118.86 ± 5.27 ^a^	3.05 ± 0.12 ^a^
*A. altissima* bark 2%	28.85 ± 1.97 ^b^	40.40 ± 1.56 ^b^	1.03 ± 0.04 ^b^
*A. altissima* branch 2%	32.35 ± 0.77 ^b^	44.41 ± 2.79 ^b^	1.08 ± 0.07 ^b^
Control EtOH	9.84 ± 0.50 ^c^	12.95 ± 1.44 ^c^	0.34 ± 0.04 ^c^
Control D. H20	4.16 ± 0.17 ^c^	6.24 ± 0.43 ^d^	0.15 ± 0.01 ^d^
*A. altissima* leaves 1%	16.99 ± 1.55 ^a^	24.19 ± 1.41 ^a^	0.56 ± 0.06 ^a^
*A. altissima* bark 1%	16.99 ± 1.58 ^a^	24.75 ± 2.15 ^a^	0.58 ± 0.06 ^a^
*A. altissima* branch 1%	18.29 ± 1.66 ^a^	26.61 ± 2.40 ^a^	0.59 ± 0.05 ^a^
Control EtOH	9.65 ± 0.38 ^b^	14.33 ± 1.17 ^b^	0.35 ± 0.02 ^b^
Control D. H20	4.30 ± 0.27 ^c^	6.10 ± 0.33 ^c^	0.15 ± 0.02 ^c^
*A. altissima* leaves 0.5%	13.81 ± 1.29 ^a^	18.56 ± 2.16 ^b^	0.43 ± 0.05 ^a^
*A. altissima* bark 0.5%	13.31 ± 1.14 ^a^	18.82 ± 1.26 ^b^	0.45 ± 0.06 ^a^
*A. altissima* branch 0.5%	14.49 ± 1.14 ^a^	21.80 ± 1.75 ^a^	0.46 ± 0.04 ^a^
Control EtOH	9.37 ± 0.60 ^b^	14.14 ± 0.36 ^c^	0.34 ± 0.03 ^b^
Control D. H20	4.08 ± 0.45 ^c^	6.21 ± 0.39 ^d^	0.14 ± 0.01 ^c^

Values are presented as means ± SD. Different lowercase letters indicate statistically significant differences among treatments at *p* < 0.05. Results are expressed as mg of Trolox equivalent/g of dry weight (mg TE/g DW). DPPH—2,2-diphenyl-1-picrylhydrazyl assay; ABTS—2,2′azino-bis (3-ethylbenzothiazoline-6-sulfonic acid)-diammonium salt) assay; FRAP—ferric-reducing antioxidant power assay.

**Table 3:** Antioxidative enzyme response of wheat seedlings to *Ailanthus altissima* treatments.

	SOD	CAT	GPX	POD	APX
*A. altissima* leaves 5%	350.12 ± 12.09 ^a^	40.68 ± 1.70 ^a^	10.1 ± 0.37 ^a^	14.10 ± 0.51 ^ab^	18.36 ± 0.81 ^b^
*A. altissima* bark 5%	222.71 ± 11.54 ^b^	39.76 ± 1.35 ^a^	9.63 ± 0.49 ^a^	13.78 ± 0.64 ^b^	18.35 ± 0.68 ^b^
*A. altissima* branch 5%	231.72 ± 2.78 ^b^	41.50 ± 1.43 ^a^	10.11 ± 0.26 ^a^	14.63 ± 0.48 ^a^	19.70 ± 0.64 ^a^
Control EtOH	48.45 ± 1.29 ^c^	20.58 ± 0.70 ^b^	4.93 ± 0.18 ^b^	7.91 ± 0.17 ^c^	9.77 ± 0.21 ^c^
Control D. H20	50.77 ± 2.08 ^c^	19.77 ± 0.89 ^b^	4.44 ± 0.10 ^b^	7.02 ± 0.31 ^c^	9.44 ± 0.38 ^c^
*A. altissima* leaves 2%	187.17 ± 3.37 ^ab^	34.66 ± 1.53 ^b^	9.31 ± 0.46 ^a^	12.91 ± 0.45 ^a^	17.36 ± 0.39 ^a^
*A. altissima* bark 2%	75.34 ± 3.30 ^b^	35.47 ± 1.58 ^ab^	8.93 ± 0.34 ^a^	12.71 ± 0.14 ^a^	17.02 ± 0.76 ^a^
*A. altissima* branch 2%	61.75 ± 2.49 ^a^	37.79 ± 1.53 ^a^	9.18 ± 0.31 ^a^	13.20 ± 0.43 ^a^	17.73 ± 0.61 ^a^
Control EtOH	49.75 ± 1.09 ^c^	19.55 ± 0.88 ^c^	4.98 ± 0.29 ^b^	6.94 ± 0.28 ^b^	9.24 ± 0.27 ^b^
Control D. H20	49.75 ± 1.78 ^c^	19.95 ± 0.86 ^c^	5.00 ± 0.11 ^b^	7.13 ± 0.30 ^b^	9.80 ± 0.28 ^b^
*A. altissima* leaves 1%	60.29 ± 2.37 ^a^	23.40 ± 1.64 ^a^	5.97 ± 0.34 ^a^	8.66 ± 0.30 ^a^	11.33 ± 0.81 ^a^
*A. altissima* bark 1%	60.12 ± 3.35 ^a^	23.75 ± 1.35 ^a^	5.83 ± 0.19 ^a^	8.08 ± 0.39 ^a^	11.51 ± 0.56 ^a^
*A. altissima* branch 1%	58.82 ± 4.34 ^a^	24.73 ± 1.11 ^a^	6.14 ± 0.28 ^a^	8.74 ± 0.58 ^a^	12.08 ± 0.80 ^a^
Control EtOH	49.47 ± 2.66 ^b^	19.69 ± 0.24 ^b^	5.05 ± 0.15 ^b^	7.07 ± 0.14 ^b^	9.77 ± 0.36 ^b^
Control D. H20	50.02 ± 0.78 ^b^	19.85 ± 1.31 ^b^	5.01 ± 0.16 ^b^	7.10 ± 0.30 ^b^	9.36 ± 0.29 ^b^
*A. altissima* leaves 0.5%	56.35 ± 3.97 ^a^	21.31 ± 1.95 ^ab^	5.77 ± 0.46 ^a^	7.62 ± 0.63 ^ab^	10.17 ± 0.87 ^ab^
*A. altissima* bark 0.5%	53.61 ± 2.29 ^ab^	21.23 ± 1.81 ^ab^	5.02 ± 0.14 ^b^	7.52 ± 0.45 ^ab^	10.02 ± 0.45 ^ab^
*A. altissima* branch 0.5%	56.12 ± 2.64 ^a^	22.90 ± 0.73 ^a^	5.77 ± 0.33 ^a^	8.20 ± 0.20 ^a^	10.87 ± 0.80 ^a^
Control EtOH	50.14 ± 2.38 ^b^	20.08 ± 0.60 ^b^	5.02 ± 0.19 ^b^	6.89 ± 0.37 ^b^	9.46 ± 0.19 ^b^
Control D. H20	50.2 ± 1.71 ^b^	20.05 ± 0.65 ^b^	5.11 ± 0.13 ^b^	6.95 ± 0.23 ^b^	9.45 ± 0.50 ^b^

Values are presented as means ± SD. Different lowercase letters indicate statistically significant differences among treatments at *p* < 0.05. Results are expressed as U/mg protein. SOD—superoxide dismutase; CAT—catalase; GPX—glutathione peroxidase; APX—ascorbate peroxidase; POD—pyrogallol peroxidase.

**Table 4:** Lipid peroxidation response of wheat seedlings to *Ailanthus altissima* extract treatments.

	Lipid Peroxidation			
	5%	2%	1%	0.50%
*A. altissima* leaves	2.50 ± 0.08 ^a^	1.98 ± 0.09 ^ab^	1.29 ± 0.13 ^a^	1.04 ± 0.15 ^a^
*A. altissima* bark	2.46 ± 0.06 ^a^	1.89 ± 0.13 ^b^	1.30 ± 0.10 ^a^	1.08 ± 0.06 ^a^
*A. altissima* branch	2.50 ± 0.16 ^a^	2.12 ± 0.12 ^a^	1.34 ± 0.12 ^a^	1.19 ± 0.08 ^a^
Control EtOH	0.99 ± 0.07 ^b^	0.94 ± 0.09 ^c^	1.04 ± 0.07 ^b^	1.06 ± 0.07 ^a^
Control D. H20	1.01 ± 0.08 ^b^	0.99 ± 0.06 ^c^	1.03 ± 0.07 ^b^	1.05 ± 0.04 ^a^

Values are presented as means ± SD. Different lowercase letters indicate statistically significant differences among treatments at *p* < 0.05. Results are expressed as μmol MDA/mg protein.

## Data Availability

The original contributions presented in this study are included in the article/Supplementary Material. Further inquiries can be directed to the corresponding author.

## Supplementary Materials

The following supporting information can be downloaded at: https://www.mdpi.com/article/10.3390/plants15081250/s1, Table S1. HPLC-DAD-MS characterization and content of phenolic compounds in *Ailanthus altissima* leaves, bark, and branches. The percentage values of the areas are the means of the 6 samples (n = 6). Table S2: Results of two-way ANOVA for the effects of plant part and extract concentration on (a) germination energy, (b) total germination, (c) shoot length, and (d) root length in wheat seedlings treated with *Ailanthus altissima* extracts.; Table S3: Results of two-way ANOVA for the effects of plant part and extract concentration on seedling vigor in wheat seedlings treated with *Ailanthus altissima* extracts.
