# Supplementary material for: Bioactivity of Ailanthus altissima (Mill.) Swingle Extracts on Wheat Germination and Rice Weevil Survival
Source: Plants (Basel). 2026 Apr 18;15(8):1250. doi: 10.3390/plants15081250 (PMC13119828; doi:10.3390/plants15081250)
Supplement: Supplementary file 1 [file plants-15-01250-s001.zip › plants-4227363-supplementary.pdf]

Table S1. HPLC-DAD-MS characterization and content of phenolic compounds in Ailanthus altissima leaves, bark, and branches. The percentage values of the areas are the mean of the 6 samples (n = 6).

Compounds	Molecular Formula	Rt HPLC-DAD (min)	λmax (nm)	Measured [M-H]-	*A. altissima* leaves	*A. altissima* bark	*A. altissima* branch
2-Hydroxybenzoic acid	$C_7H_6O_3$	3.19	270	139	2.926	0.201	0.988
4-Caffeoyquinic acid (Cryptochlorogenic acid)	$C_{16}H_{18}O_9$	10.07	332	355	10.253	0	0.089
3-Caffeoyquinic acid (Neochlorogenic acid)	$C_{16}H_{18}O_9$	10.77	332	355	17.221	0.031	0.221
5-Caffeoyquinic acid (Chlorogenic acid)	$C_{16}H_{18}O_9$	12.55	332	355	14.414	0.032	0.381
Punicalin	$C_{34}H_{22}0_{22}$	13.72	280	783	4.138	0.368	0.686
p-Coumaroylquinic acid	$C_{16}H_{18}O_8$	14.38	333	339	0.974	0	0.129
Quercetin-rutinoside (Rutin)	$C_{27}H_{30}O_{16}$	15.73	360, *260*	611, *303*	6.444	0.036	0.146
Quercetin-glucoside	$C_{21}H_{20}O_{12}$	16.34	360, *260*	465, *303*	21.445	0.088	0.627
Ferulic acid	$C_{10}H_{10}O_4$	16.85	334	195	0	0.589	0.288
Quercetin-malonyl-glucoside	$C_{24}H_{22}O_{15}$	17.19	360, *260*	551, *303*	12.608	0.083	0
Kaempferol-glucoside	$C_{21}H_{20}O_{11}$	17.42	350, *250*	449, *287*	5.882	0.05	0.27
Apigenin-galactoside	$C_{21}H_{20}O_{10}$	17.74	340, *260*	433, *271*	3.064	0.017	0.053
Luteolin-malonyl-glucoside	$C_{24}H_{22}0_{14}$	18.09	350, *260*	535, *287*	1.351	0.012	0.097
Kaempferol-malonyl-glucoside	$C_{24}H_{22}O_{14}$	18.59	350, *250*	535, *287*	4.258	0	0
IsoFerulic acid	$C_{10}H_{10}O_4$	18.76	334	195	0	0.251	0.642
Luteolin	$C_{15}H_{10}O_6$	21.46	350, *260*	287	0.14	0.014	0.017
**TOTAL PHENOLICS**					**105.118**	**1.772**	**4.635**

Table S2. Results of two-way ANOVA for the effects of plant part and extract concentration on a) germination energy, b) total germination, c) shoot length, and d) root length in wheat seedlings treated with *Ailanthus altissima* extracts.

a) Germination energy

Source	df	Sum of squares	Mean square	F value	P-value
Corrected model	11	2593.733	235.794	55.699	<0.001
Intercept	1	309889.067	309889.067	73202.142	<0.001
Plant part	2	1565.233	782.617	184.87	<0.001
Concentration	3	175.6	58.533	13.827	<0.001
Plant part × Concentration	6	852.9	142.15	33.579	<0.001
Error	48	203.2	4.233		
Total	60	312686			

b) Total germination

Source	df	Sum of squares	Mean square	F value	P-value
Corrected model	11	2996.6	272.418	57.859	<0.001
Intercept	1	381125.4	381125.4	80946.988	<0.001
Plant part	2	2062.9	1031.45	219.069	<0.001
Concentration	3	139.267	46.422	9.86	<0.001
Plant part × Concentration	6	794.433	132.406	28.122	<0.001
Error	48	226	4.708		
Total	60	384348			

c) Shoot length

Source	df	Sum of squares	Mean square	F value	P-value
Corrected model	11	497.783	45.253	26.751	<0.001
Intercept	1	14446.017	14446.017	8539.517	<0.001
Plant part	2	327.633	163.817	96.837	<0.001
Concentration	3	10.183	3.394	2.007	0.126
Plant part × Concentration	6	159.967	26.661	15.76	<0.001
Error	48	81.2	1.692		
Total	60	15025			

d) Root length

Source	df		Sum of squares	Mean square	
Corrected model	11	503.533	45.776	F value	P-value
Intercept	1	7041.667	7041.667	15.605	<0.001
Plant part	2	213.433	106.717	2400.568	<0.001
Concentration	3	40.733	13.578	36.381	<0.001
Plant part × Concentration	6	249.367	41.561	4.629	0.006
Error	48	140.8	2.933	14.169	<0.001
Total	60	7686			

Table S3. Results of two-way ANOVA for the effects of plant part and extract concentration on seedling vigor in wheat seedlings treated with *Ailanthus altissima* extracts.

Seedling Vigor Index

**Source**	**df**	**Sum of squares**	**Mean square**	**F value**	**P-value**
Corrected model	11	5737860	521623.6	27.73	<0.001
Intercept	1	48386036	48386036	2572.28	<0.001
Plant part	2	2962713	1481357	78.751	<0.001
Concentration	3	225608.1	75202.68	3.998	0.013
Plant part × Concentration	6	2549539	424923.1	22.59	<0.001
Error	48	902907.2	18810.57		
Total	60	55026803			

Table S4. Results of two-way ANOVA for the effects of plant part and extract concentration on a) total phenolic, b) total tannins, and c) total flavonoids contents in wheat seedlings treated with *Ailanthus altissima* extracts.

a) Total phenolics

Source	df	Sum of squares	Mean square	F value	P-value
Corrected model	11	1486.31	135.119	300.867	<0.001
Intercept	1	8818.301	8818.301	19635.57	<0.001
Plant part	2	290.322	145.161	323.228	<0.001
Concentration	3	966.443	322.148	717.321	<0.001
Plant part × Concentration	6	229.544	38.257	85.187	<0.001
Error	60	26.946	0.449		
Total	72	10331.56			

b) Total tannins

Source	df	Sum of squares	Mean square	F value	P-value
Corrected model	11	723.471	65.77	394.821	<0.001
Intercept	1	3338.289	3338.289	20039.93	<0.001
Plant part	2	35.613	17.806	106.892	<0.001
Concentration	3	572.329	190.776	1145.241	<0.001
Plant part × Concentration	6	115.529	19.255	115.588	<0.001
Error	60	9.995	0.167		
Total	72	4071.755			

c) Total flavonoids

Source	df	Sum of squares	Mean square	F value	P-value
Corrected model	11	748.597	68.054	401.929	<0.001
Intercept	1	3341.913	3341.913	19737.37	<0.001
Plant part	2	41.119	20.559	121.425	<0.001
Concentration	3	584.761	194.92	1151.202	<0.001
Plant part × Concentration	6	122.717	20.453	120.795	<0.001
Error	60	10.159	0.169		
Total	72	4100.669			

Table S5. Results of two-way ANOVA for the effects of plant part and extract concentration on antioxidant activity parameters estimated by a) DPPH, b) ABTS, and c) FRAP assays in wheat seedlings treated with *Ailanthus altissima* extracts.

a) DPPH

Source	df	Sum of squares	Mean square	F value	P-value
Corrected model	11	267042.1	24276.56	1009.44	<0.001
Intercept	1	174527.1	174527.1	7256.988	<0.001
Plant part	2	90764.78	45382.39	1887.039	<0.001
Concentration	3	82979.64	27659.88	1150.122	<0.001
Plant part × Concentration	6	93297.71	15549.62	646.567	<0.001
Error	60	1442.971	24.05		
Total	72	443012.2			

b) ABTS

Source	df	Sum of squares	Mean square	F value	P-value
Corrected model	11	104003.4		956.596	<0.001
Intercept	1	165304.6	9454.853	16724.72	<0.001
Plant part	2	26780.96	165304.6	1354.784	<0.001
Concentration	3	48019.75	13390.48	1619.468	<0.001
Plant part × Concentration	6	29202.66	16006.59	492.431	<0.001
Error	60	593.031	4867.11		
Total	72	269901.1	9.884		

c) FRAP

Source	df	Sum of squares	Mean square	F value	P-value
Corrected model	11	72.685		886.165	<0.001
Intercept	1	103.598	6.608	13893.62	<0.001
Plant part	2	18.163	103.598	1217.941	<0.001
Concentration	3	35.267	9.082	1576.539	<0.001
Plant part × Concentration	6	19.255	11.756	430.387	<0.001
Error	60	0.447	3.209		
Total	72	176.731	0.007		

Table S6. Results of two-way ANOVA for the effects of plant part and extract concentration on the activities of antioxidant enzymes a) SOD, b) CAT, c) GPX, d) POD, and e) APX in wheat seedlings treated with *Ailanthus altissima* extracts.

a) SOD

Source	df	Sum of squares	Mean square	F value	P-value
Corrected model	11	614636.1	55876.01	1474.378	<0.001
Intercept	1	999850.7	999850.7	26382.66	<0.001
Plant part	2	15541.89	7770.946	205.049	<0.001
Concentration	3	553804.5	184601.5	4871.006	<0.001
Plant part × Concentration	6	45289.72	7548.286	199.174	<0.001
Error	60	2273.881	37.898		
Total	72	1616761			

b) CAT

Source	df	Sum of squares	Mean square	F value	P-value
Corrected model	11	4578.102	416.191	152.41	<0.001
Intercept	1	67412.54	67412.54	24686.63	<0.001
Plant part	2	46.129	23.064	8.446	<0.001
Concentration	3	4520.908	1506.969	551.856	<0.001
Plant part × Concentration	6	11.065	1.844	0.675	0.67
Error	60	163.844	2.731		
Total	72	72154.48			

c) GPX

Source	df	Sum of squares	Mean square	F value	P-value
Corrected model	11	270.771	24.616	171.913	<0.001
Intercept	1	4208.634	4208.634	29392.8	<0.001
Plant part	2	3.129	1.564	10.926	<0.001
Concentration	3	266.889	88.963	621.312	<0.001
Plant part × Concentration	6	0.753	0.125	0.876	0.518
Error	60	8.591	0.143		
Total	72	4487.996			

d) POD

Source	df	Sum of squares	Mean square	F value	P-value
Corrected model	11	553.157	50.287	199.276	<0.001
Intercept	1	8468.73	8468.73	33559.7	<0.001
Plant part	2	5.47	2.735	10.838	<0.001
Concentration	3	546.965	182.322	722.5	<0.001
Plant part × Concentration	6	0.722	0.12	0.477	0.823
Error	60	15.141	0.252		

Total	72	9037.028			

e) APX

Source	df	Sum of squares	Mean square	F value	P-value
Corrected model	11	951.127	86.466	147.835	<0.001
Intercept	1	15224.76	15224.76	26030.38	<0.001
Plant part	2	11.08	5.54	9.472	<0.001
Concentration	3	938.102	312.701	534.637	<0.001
Plant part × Concentration	6	1.945	0.324	0.554	0.765
Error	60	35.093	0.585		
Total	72	16210.98			

Table S7. Results of two-way ANOVA for the effects of plant part and extract concentration on lipid peroxidation in wheat seedlings treated with *Ailanthus altissima* extracts.

Lipid peroxidation

**Source**	**df**	**Sum of squares**	**Mean square**	**F value**	**P-value**
Corrected model	11	22.13	2.012	134.617	<0.001
Intercept	1	213.935	213.935	14315.061	<0.001
Plant part	2	0.14	0.07	4.7	0.013
Concentration	3	21.884	7.295	488.116	<0.001
Plant part × Concentration	6	0.105	0.018	1.174	0.332
Error	60	0.897	0.015		
Total	72	236.961			

Table S8. Repeated measurements ANOVA of mortality measured at 24, 48, and 72 h after treatment.

Source	df	Sum of squares	Mean square	F value	P-value
Plant part	4	5019	1255	59.8	<0.001
Concentration	3	3746	1249	59.5	<0.001
Plant part*Concentration	12	3483	290	13.8	<0.001
DAYS	2	1320	660	133.9	<0.001
DAYS*Plant part	8	1082	135	27.4	<0.001
DAYS*Concentration	6	877	146	29.6	<0.001
DAYS*Plant part*Concentration	24	925	39	7.8	<0.001

Table S9. Repeated measurements ANOVA for progeny production across 30, 60, and 90 days.

**Source**	**df**	**Sum of squares**	**Mean square**	**F value**	**P-value**
Plant part	4	6636645	1659161	923.25	<0.001
Concentration	3	1849063	616354	342.97	<0.001
Plant part*Concentration	12	1613249	134437	74.81	<0.001
DAYS	2	4097791	2048896	1964.58	<0.001
DAYS*Plant part	8	1846180	230772	221.28	<0.001
DAYS*Concentration	6	23170	3862	3.70	0.0026
DAYS*Plant part*Concentration	24	128476	5353	5.13	<0.001
